# Validation of a single question for assessment of habitual physical activity in women

**DOI:** 10.1186/s41687-025-00986-y

**Published:** 2025-12-21

**Authors:** Melissa J. Benton, Andrea M. Hutchins

**Affiliations:** Helen and Arthur E. Johnson Beth-El College of Nursing & Health Sciences, University of Colorado Colorado Springs, 1420 Austin Bluffs Parkway, Colorado Springs, 80918 USA

**Keywords:** Construct validity, Convergent validity, Known-groups validity, Self-report, Quality of life

## Abstract

**Background:**

Accurate assessment of physical activity behaviors allows clinicians to target counseling more effectively. Single questions regarding habitual physical activity provide the most feasible option for rapid physical activity assessment. This study evaluated construct validity of a single item physical activity question to self-report habitual activity as simple categorical descriptors (*not active*,* somewhat active*,* active*,* very active*).

**Methods:**

Women (*N* = 120) completed one laboratory session for measurement of self-reported physical activity (International Physical Activity Questionnaire-IPAQ), anthropometrics and body composition, and physical activity-related quality of life (SF-36 physical function subscale, Vitality Plus Scale). Construct validity was evaluated as convergent and known-groups validity.

**Results:**

Mean age was 60 ± 16 years (range 25–89). The majority (62%) reported being *active* or *very active.* Age was not significantly related to activity levels. Correlation analysis demonstrated good convergent validity. Significant negative correlations were found with body weight, body mass index (BMI), waist circumference, and body fat (all *p* <0.001). Significant positive correlations were found with vigorous intensity activity (*p* <0.001), moderate intensity activity (*p* = .004), walking (*p* = .005), and quality of life (*p* <0.001). Good known-groups validity was demonstrated by significant differences between habitual physical activity levels for body weight, waist circumference, body fat, vigorous intensity activity (all *p* <0.001), moderate intensity activity (*p* = .038), walking (*p* = .049), and quality of life (*p* <0.001). Regression models confirmed known-groups validity.

**Conclusions:**

A single question with categorical descriptors is valid for brief clinical assessment of habitual physical activity in women across a wide age range.

## Background

The evidence that physical activity is beneficial and promotes health is conclusive. Among community-dwelling adults, regular physical activity at or above published guidelines [1] can prevent long-term weight gain [2] and the onset of chronic diseases such as breast cancer, colon cancer, diabetes, macular degeneration, and heart disease [3–6]. Participation in moderate-vigorous physical activity decreases overall mortality risk among adults [7] and older adults [8]. It can also decrease risk for mortality related specifically to cardiovascular disease, cancer, and diabetes [9, 10]. Physical activity also promotes mental health [11] and reduces the risk for depression [12, 13]. Finally, there is evidence that moderate-vigorous physical activity can decrease risk for dementia [14, 15] and may improve cognitive function in older adults [16]. Most importantly, physical activity is safe and the health benefits outweigh the risks, which are extremely low [17, 18].

Physical activity levels among all age groups have decreased significantly over the past two decades [19, 20]; and over the lifespan, females are observed to have lower physical activity levels compared to males [21–23]. Insufficient physical activity places women at risk for chronic diseases including heart failure [24], cancer [25], type 2 diabetes [4], long COVID [26], and dementia [27], in addition to cardiovascular and all-cause mortality [28]. Furthermore, women experience risk for sarcopenia [29, 30], frailty [31], loss of physical function and independence [32], and diminished quality of life [33–35] due to low physical activity.

Physical activity counseling by healthcare providers is recommended in primary care settings to promote cardiovascular and metabolic health [36–38]. Accurate assessment of physical activity behaviors allows clinicians to target counseling more effectively. However, direct physical activity assessment using accelerometers or pedometers can be overly burdensome, requiring multiple days of measurement [39]. Accuracy can also be a consideration of direct measurement technologies. Accelerometers and pedometers rely on proprietary algorithms for calculation of activity and so may not be accurate for all populations [40]. Accuracy may also be diminished by the positioning of devices on the body and variations in exercise intensity [40].

Self-reported measures of physical activity provide alternatives to direct measurement that are relatively easy to administer and less burdensome. The International Physical Activity Questionnaire (IPAQ) is the most frequently used and most widely validated self-report of physical activity [41, 42]. However, even the IPAQ short form (IPAQ-SF), which includes seven open-ended questions, requires 5–10 min for completion and additional time for scoring, making it impractical for healthcare settings. Furthermore, adult respondents report difficulties classifying activities into intensity categories and calculating frequencies and durations in response to the open-ended questions [43, 44].

By comparison, single questions regarding habitual physical activity may provide the most feasible option for rapid physical activity assessment. Single questions are brief, easy to administer, easy to score, and can be completed in less than one minute [45]. Single questions have been validated against accelerometry [46–48], physical performance [47, 49], anthropometrics [49], and the IPAQ [50]. However, these questions can still require respondents to quantify frequency, intensity, and duration of activities [46–48, 50], which creates the same burden as previously noted with the IPAQ. These single questions ask participants to quantify the frequency of their physical activity descriptively (*never*,* occasionally*,* few times a week*,* almost daily*) instead of numerically [50], or respondents are asked to use a complex definition of “physical activity” (*a total of 30 min or more of physical activity*,* which was enough to raise your breathing rate*) [48]. Single questions have also used multiple time periods for recall including *the past week* [48] and *the past six months* [46]. Another single question asks respondents to characterize their activity using seven complex response options with varying frequency, intensity, and activity types [47]. An alternative format has been reported in which a single question asks respondents to characterize their habitual activity using categorical descriptors (*not active*,* somewhat active*,* active*,* very active*) that can be converted to a Likert scale [49]. This approach avoids the need to quantify frequency, intensity, and duration of either current or past physical activity.

Use of categorical descriptors to replace quantification is a promising approach to brief physical activity assessment. A single question that categorizes habitual physical activity can be used for efficient assessment and delivery of targeted education by healthcare providers, but further validation is needed. Therefore, the purpose of this study was to evaluate construct validity for a single item physical activity question that allows women to self-report their habitual activity as simple categorical descriptors. This question has previously been validated in a single sample of older women [49], so validation in women with a wider age range is needed. No hypotheses were specified prior to study implementation.

## Methods

### Participants

Women were recruited from the community in the state of Colorado, USA using emails, flyers, and word of mouth, and to diminish burden were screened by telephone for eligibility. They were included in the study if they were 25 years or older and did not currently smoke any tobacco product. The only exclusion criterion was failure to follow pre-testing diet and exercise restrictions. One participant was excluded for failure to follow diet restrictions prior to measurement. The study was approved by the university institutional review board and women signed an informed consent before participation in data collection.

### Design and data collection

This was a cross-sectional study. All testing was completed in the same laboratory setting by the two principal investigators. Data were collected in the morning and participants were asked to adhere to the following diet and exercise restrictions prior to testing: no food for at least four hours, no caffeinated beverages for at least eight hours, and no exercise for 24 h. Water consumption was encouraged *ad libitum* to promote hydration. Measurements included objective and subjective variables that have previously been demonstrated to be related to or influenced by physical activity.

### Self-reported physical activity

Women answered a single question regarding habitual physical activity levels (*How would you rate your lifestyle?*) by choosing one of four response options (*Not active*,* Somewhat active*,* Active*,* Very active*). No specific instructions were given to participants regarding the meaning of “lifestyle” or a recall period for completion of the question. Responses were converted to a four-point Likert-like scale from 0-*Not active* to 3-*Very active*. This question has been previously validated for use among community-dwelling older women and found to be significantly correlated with self-reported moderate (*r*_s_ = 0.46) and vigorous (*r*_s_ = 0.53) exercise [49].

Participants then completed the IPAQ-SF [42, 51] for self-reported activity during the last seven days. The IPAQ-SF includes six items in which respondents are asked to quantify frequency (days per week) and duration (hours/minutes per day) of vigorous activity, moderate activity, and walking. Physical activity was calculated as: frequency (number of days) X duration (minutes per day) X METs for each activity level. METs (metabolic equivalents) were assigned based on recommended scoring guidelines as 3.3 METs for walking, 4.0 METs for moderate intensity, and 8.0 METs for vigorous intensity [52]. Individual activity levels (walking, moderate, vigorous) were reported as MET-minutes/week.

### Anthropometric and body composition measures

Height was measured with a wall-mounted stadiometer and body weight was measured with an electronic digital scale (Tanita Corp., USA). Women were asked to void prior to measurement and both measures were taken without shoes. Waist circumference was measured at the level of the umbilicus using a Gulick tape measure to control tension.

Body composition was measured with multifrequency bioelectrical impedance analysis (Quadscan 4000, Bodystat, UK). Before testing, adequate hydration was verified with urine specific gravity (Accutest, Jant, USA), and test electrodes were placed on the right hand and foot using standardized anatomical markers for positioning [53]. Participants then rested quietly in a supine position for at least 5 min. Body fat was calculated by the Quadscan device using proprietary equations [54] and reported as relative (%) fat mass.

### Physical activity-related quality of life

The physical function subscale of the Short Form-36 (SF-36) was used to measure quality of life related to physical activity. The SF-36 has been validated and widely used for measurement of quality of life in adults [55, 56]. The physical function subscale includes 10 items regarding functional ability and participation in physical activities that participants rated on a 3-point Likert scale (*limited a lot*,* limited a little*,* not limited at all*). For analysis, responses were converted to numeric scores (0, 50, 100), with higher scores indicating better physical activity-related quality of life.

Subjective benefits related to exercise participation were measured with the Vitality Plus Scale. The Vitality Plus Scale is a brief, 10-item tool that has been validated as a measure of exercise-related health outcomes in adults [57]. Each item was rated on a scale from 1 to 5, with higher scores indicating greater health benefits. Scores for each item were totaled for a summative score ranging from 10 to 50.

### Sample size calculation

Sample size was calculated based on previously reported correlations (*r*_s_ = 0.46–0.53) between the single item question and self-reported moderate and vigorous activity [49]. Using the most conservative estimate of *r*_s_ = 0.46, with one-sided alpha (*p* = .05) and 90% power (β = 0.10), an adequate sample size was at least 90 participants [58].

### Statistical analysis

Data were analyzed using SPSS version 29 (IBM Corp., USA). Habitual physical activity levels were found to be non-normally distributed so non-parametric tests were used for analysis. Construct validity was evaluated as convergent and known-groups validity. Convergent validity was analyzed using Spearman rank correlation analysis to identify relationships between habitual physical activity levels and variables of interest. For known-groups validity, a Kruskal-Wallis test was used to analyze differences between habitual physical activity levels. When significant differences were identified, Dunn’s test was used for post hoc comparisons. To confirm known-groups validity, outcome variables were entered into linear regression models to determine the predictive ability of the single question response options. For all analyses, results were considered to be statistically significant at the *p* <0.05 level. Descriptive data were reported as frequencies (%) or means (standard deviations) with 95% confidence intervals and medians [interquartile range].

## Results

A total of 120 women participated in the study. Overall, the sample was predominantly White (82%) and ranged in age from 25 to 89 years. Age was not found to be significantly related to habitual physical activity levels (*r*_*s*_ = 0.08, *p* = .360). As a single group, participants were overweight with a BMI of 27.1 ± 5.9 kg/m^2^ and body fat of 39.4 ± 7.5%. The majority (62%) reported being either *active* or *very active*. Participant characteristics are summarized in Table 1.

**Table 1:** Characteristics of the entire sample (*N* = 120)

Characteristics	*n* (%)	
Physical Activity Level		
*Not active* *Somewhat active* *Active* *Very active*	6 (5) 39 (33) 54 (45) 21 (17)	
Race/Ethnicity		
White Black Hispanic Asian Hispanic/Black American Indian Not reported	98 (82) 8 (7) 9 (8) 1 (1) 2 (2) 1 (1) 1 (1)	

	Mean (SD)	Median [IQR]
Age (years)	60.2 (15.9)	63.5 [53–71]
Height (cm)	162.8 (6.7)	162.5 [158–167]
Weight (kg)	71.8 (15.7)	69.4 [63-78.7]
Waist circumference (cm)	88.4 (13.3)	88 [78-96.8]
Body mass index (kg/m^2^)	27.1 (5.9)	26.1 [23.2–30.9]
Fat mass (%)	39.4 (7.5)	39.6 [34-45.3]
Vigorous activity (MET-minutes/week)	1533 (1875)	1080 [0-2160]
Moderate activity (MET-minutes/week)	1249 (1453)	720 [210–1680]
Walking (MET-minutes/week)	1465 (1593)	792 [396–2079]
Physical function (SF-36 subscale score)	87.3 (19.6)	95 [85–100]
Vitality Plus Scale (score)	36.1 (6.9)	37 [31.3–42]

Data reported as frequency (%) or mean (standard deviation) with 95% confidence intervals and median [interquartile range]

MET = metabolic equivalents

SF-36 = Short Form-36

### Convergent validity

Statistically significant correlations were identified between self-reported habitual physical activity levels and both objective and subjective variables of interest (Table 2). Specifically, moderate negative correlations were identified between habitual physical activity levels and body weight, BMI, waist circumference, and body fat. By comparison, weak positive correlations were identified with vigorous intensity activity, moderate intensity activity, walking, and physical activity-related quality of life. Although modest in strength, the directions of these correlations support the validity of the single question. Negative correlations indicate that as habitual activity increases, body weight, BMI, waist circumference, and fat mass decrease. Positive correlations indicate that as habitual activity increases, duration and frequency of vigorous, moderate, and walking activities, and activity-related quality of life increase.

**Table 2:** Convergent validity of self-reported habitual physical activity levels (*N* = 120)

	BW	BMI	WC	FM	VIG	MOD	WLK	PF	VPS
**PA**	− 0.449 *p* <0.001	− 0.456 *p* <0.001	− 0.492 *p* <0.001	− 0.447 *p* <0.001	0.390 *p* <0.001	0.265 *p* = .004	0.255 *p* = .005	0.361 *p* <0.001	0.387 *p* <0.001

Data reported as Spearman’s rho (*r*_*s*_); *p*-values

PA = physical activity levels, BW = body weight, WC = waist circumference, BMI = body mass index, FM = % fat mass, VIG = vigorous intensity activity, MOD = moderate intensity activity, WLK = walking, PF = physical function subscale from the SF-36, VPS = Vitality Plus Scale

### Known-Groups validity

**Fig. 1:**
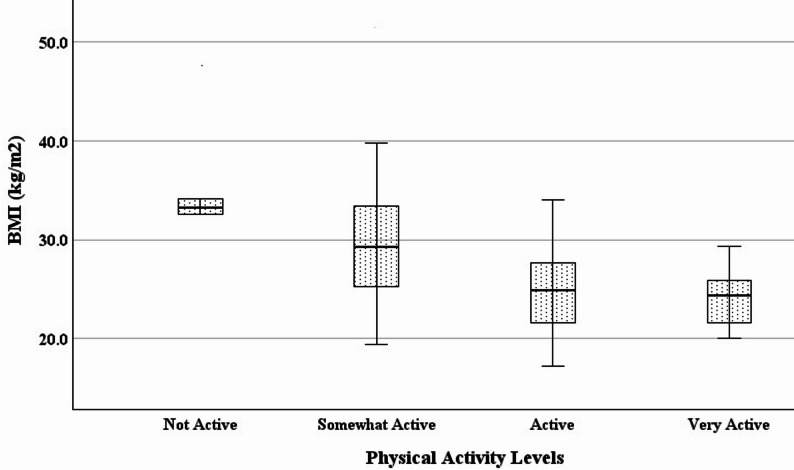
Decreases in BMI (kg/m^2^) across habitual physical activity levels. (*N* = 120; *p* <0.001). BMI = body mass index

**Fig. 2:**
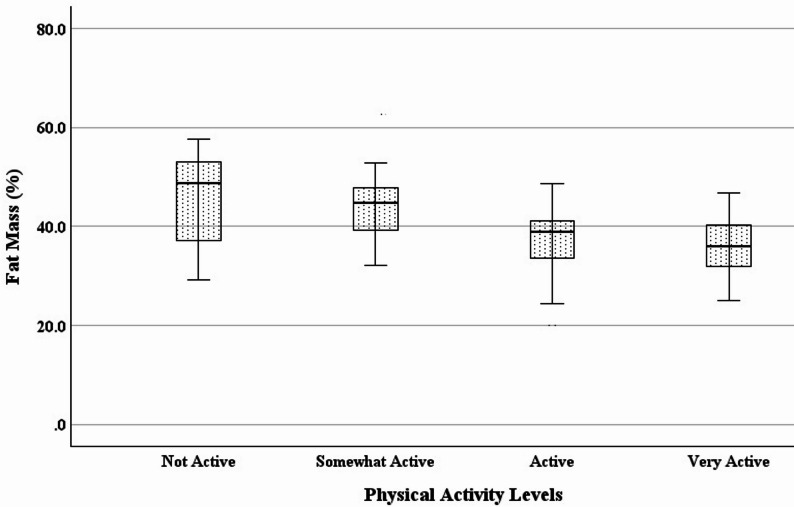
Decreases in % body fat across habitual physical activity levels. (*N* = 120; *p* <0.001)

**Fig. 3:**
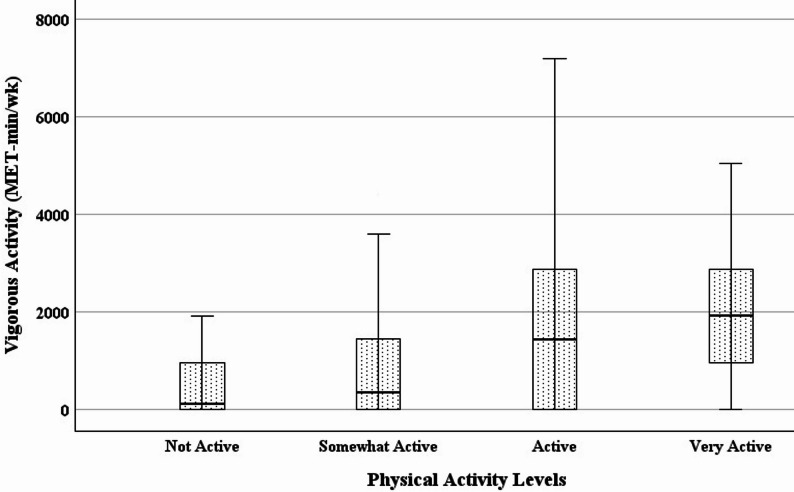
Increases in vigorous physical activity across habitual physical activity levels. (*N* = 120; *p* <0.001)

**Fig. 4:**
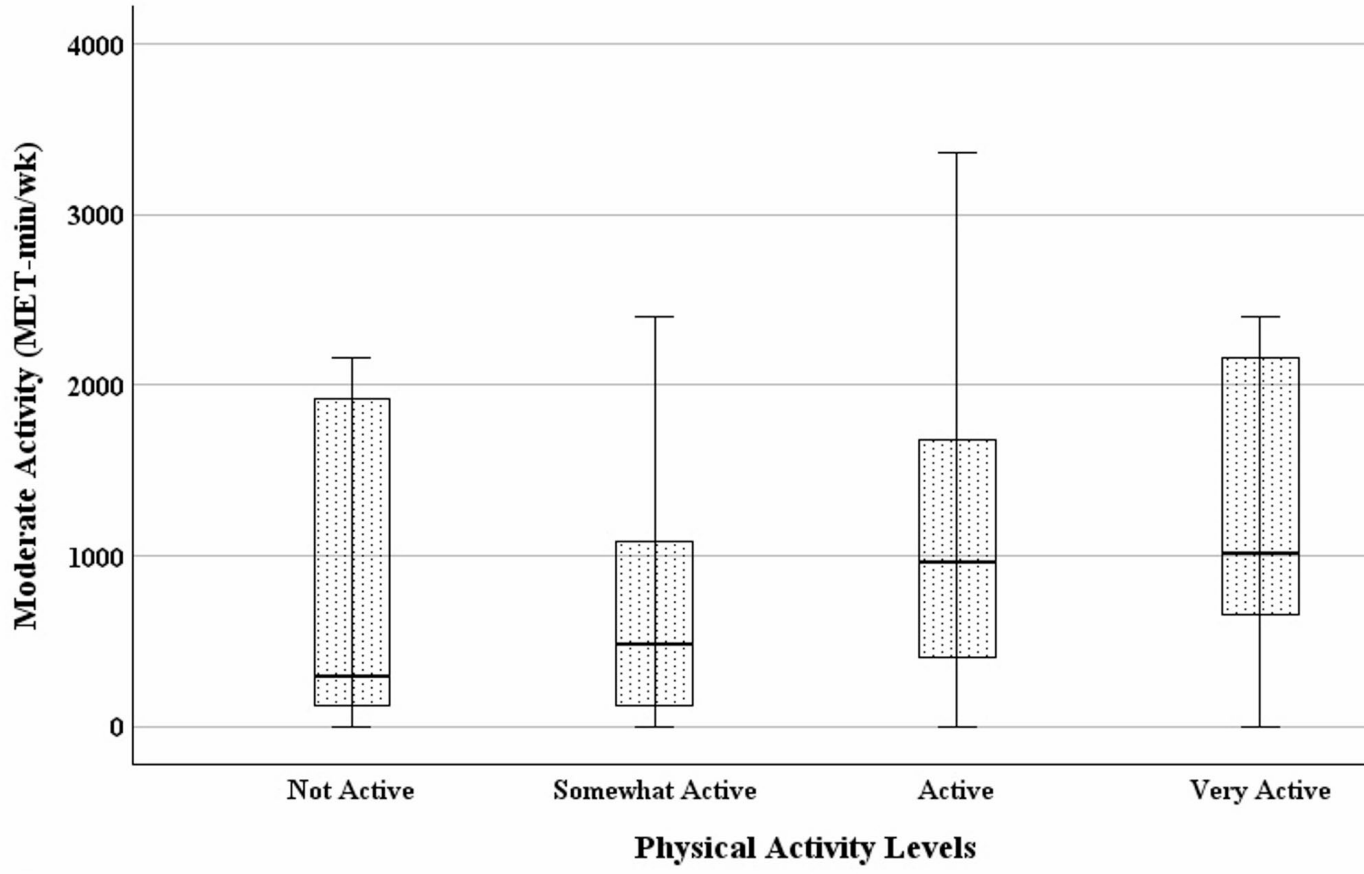
Increases in moderate physical activity across habitual physical activity levels. (*N* = 120; *p* = .038)

Statistically significant differences were found between subgroups of habitual physical activity levels (Table 3; Figs. 1, 2, 3 and 4). Specifically with increasing activity levels, ranked subgroups had progressively lower body weight, BMI, waist circumference, and fat mass, and progressively greater vigorous activity, moderate activity, walking, and physical activity-related quality of life. These subgroup differences reflect a strong and consistent ability of the single question to discriminate between objectively and subjectively measured variables associated with physical activity. Participants reporting higher levels of habitual physical activity had lower body weight, BMI, waist circumference, and fat mass, and reported greater hours and frequency of vigorous, moderate, and walking activity, as well as quality of life.

**Table 3:** Known-groups validity of habitual physical activity levels (*N* = 120)

Characteristics	Not Active	Somewhat Active	Active	Very Active	H-statistic *p*-value
Weight (kg)	91.4 (26.5)	79.8 (16.3)	66.7 (10.7)*^	64.3 (11.0)*^	*H*(3) = 25.582 *p* <0.001
Waist circumference (cm)	106.2 (19.5)	95.2 (11.0)	84.7 (11.4)*^	80.0 (9.3)*^	*H*(3) = 29.424 *p* <0.001
Body mass index (kg/m^2^)	34.3 (7.6)	30.3 (6.5)	25.2 (4.1)*^	24.3 (3.5)*^	*H*(3) = 26.922 *p* <0.001
Fat mass (%)	45.8 (10.6)	43.4 (6.7)	37.3 (6.5)*^	35.5 (5.9)*^	*H*(3) = 24.982 *p* <0.001
Vigorous activity (MET-minutes/week)	523 (779)	800 (1105)	1753 (1955)**^**	2617 (2367)*^	*H*(3) = 18.108 *p* <0.001
Moderate activity (MET-minutes/week)	800 (971)	896 (1254)	1346 (1461)	1812 (1755)**^**	*H*(3) = 8.425 *p* = .038
Walking (MET-minutes/week)	866 (1355)	1243 (1728)	1564 (1593)	1804 (1376)**^**	*H*(3) = 7.866 *p* = .049
Physical function (SF-36 subscale score)	70.8 (26.0)	85.5 (16.2)	86.9 (22.9)	96.4 (7.3)*	*H*(3) = 15.902 *p* = .001
Vitality Plus Scale (score)	27.9 (5.6)	34.5 (6.7)*	36.5 (6.0)*	40.2 (6.8)*^#	*H*(3) = 21.287 *p* <0.001

Data reported as mean (standard deviation); Kruskal-Wallis *H*-statistic; *p*-value

MET = metabolic equivalents

SF-36 = Short Form-36

*Significantly different than not active

^Significantly different than somewhat active

#Significantly different than active

Regression analysis confirmed known-groups validity. A one-level increase in habitual physical activity predicted a decrease in BMI of 3.5 kg/m^2^ (*F*(1, 118) = 34.949, *p* <0.001, *R*^*2*^ = 0.229), a decrease in waist circumference of 8.6 cm (*F*(1, 118) = 43.257, *p* <0.001, *R*^*2*^ = 0.268), and a decrease in body fat of 4.1% (*F*(1, 118) = 28.617, *p* <0.001, *R*^*2*^ = 0.195). Also, a one-level increase in habitual physical activity predicted an increase in vigorous activity of 830 MET-minutes/week (*F*(1, 118) = 16.993, *p* <0.001, *R*^*2*^ = 0.126), an increase in moderate activity of 406 MET-minutes/week (*F*(1, 116) = 6.108, *p* = .015, *R*^*2*^ = 0.050), an increase in the SF-36 physical function subscale of 6.0 points (*F*(1, 118) = 7.577, *p* = .007, *R*^*2*^ = 0.060), and an increase in the Vitality Plus Scale of 3.2 points (*F*(1, 118) = 19.791, *p* <0.001, *R*^*2*^ = 0.144).

## Discussion

The current study provides support for use of a brief and easy-to-administer single question for assessment of habitual physical activity among women. Both convergent and known-groups validity were demonstrated, indicating that the categorical response options not only accurately reflect habitual physical activity levels, but also that the different levels of habitual activity (*not active*,* somewhat active*,* active*,* very active*) accurately reflect differences in participant characteristics related to participation in physical activity.

In the current study, the IPAQ-SF was used to demonstrate that the four categorical levels of habitual activity were related to and had the ability to discriminate between varying intensities and volumes of activity. When initially validated, the IPAQ-SF demonstrated good agreement with accelerometry for total physical activity over the previous 7 days, and agreement was even stronger for greater volumes and intensities of activity [51]. This mirrors our current findings of somewhat stronger correlations with progressively greater intensities of activity. In fact, the correlations we observed between physical activity levels reported with the single question and the IPAQ-SF (Table 2) are similar to the correlations between self-reported total physical activity over the past seven days measured with the IPAQ-SF and accelerometry-measured physical activity (*r*_s_ = 0.26–0.40) that were originally used for validation of the IPAQ-SF [51]. Furthermore, since initially validated, subsequent studies have demonstrated significant correlations between objective physical activity measurement with accelerometry and vigorous intensity activity, moderate intensity activity, and walking measured with the IPAQ-SF [42]. Although we recognize that the IPAQ is not a substitute for objective measurement, recent meta-analyses have reported acceptable agreement with accelerometry for vigorous, moderate, and walking activities [59]. Moreover, the correlations currently observed between the single question habitual activity levels and IPAQ-SF (Table 2) are similar to overall weighted mean correlations between accelerometry and IPAQ-SF moderate (*r*_w_ = 0.15) and vigorous (*r*_w_ = 0.48) activity that were calculated through meta-analysis [59].

In women, habitual physical activity with or without caloric restriction decreases body weight [60] and is associated with long-term maintenance of healthy weight [61]. In addition to body weight we included BMI as one of our variables of interest. BMI is sensitive to habitual physical activity, with more active women demonstrating lower BMI compared to those who are less active [62]. Based on the observed differences in BMI between self-reported levels of habitual physical activity, women in the current study who reported being *very active* also most closely approximated the BMI of 21 kg/m^2^ that has been identified as having the lowest long-term mortality risk for women [63]. It should also be noted that the predicted difference in BMI of -3.5 kg/m^2^ with a one-level increase in habitual physical activity approximates the − 5 kg/m^2^ reduction in BMI that has been found to reduce overall mortality by 20% [64]. Finally, based on the mean BMI of our sample (27.1 kg/m^2^), the predicted decrease in BMI with a one-level increase in habitual physical activity would be sufficient to move average BMI into the “healthy” range of ≤ 24.9 kg/m^2^ for the women in our study.

Waist circumference is considered a marker of both body composition and health [65, 66]. Physical activity, especially at higher intensities, is negatively associated with waist circumference [67], and habitual physical activity decreases waist circumference in women [68]. Notably, the average waist circumferences for the *not active* and *somewhat active* women in our study exceeded the threshold value of 88 cm recommended for health [69], while those for the *active* and *very active* women did not. Women with high waist circumferences that exceed the 88 cm threshold are at greater risk for hypertension, hypercholesterolemia, type 2 diabetes, and metabolic syndrome [70]. Furthermore, the predicted decrease in waist circumference of -8.6 cm in response to a one-level increase in habitual physical activity exceeds the minimal clinically important difference of -2 cm previously calculated for waist circumference [71], as well as the.

-5 cm difference found to be associated with a 9% reduction in mortality risk in women [65].

Relative (%) body fat is also sensitive to physical activity, especially at higher intensities [68]. Although absolute (kg) body fat is frequently evaluated as an outcome of physical activity interventions [68], we chose to normalize fat mass as a percentage of body mass in order to provide what we believe to be a more precise comparison between groups. The between-group differences in percent body fat we observed are consistent with differences obtained through physical activity interventions [72]. Additionally, we would argue that the 4.1% decrease in body fat predicted by a one-level increase in self-reported habitual physical activity is consistent with and likely more meaningful than the 5% reduction in total body weight that has been recommended for health promotion [73], especially given that when weight loss occurs, loss of lean mass can represent more than 10% of the total weight lost [74].

In addition to objectively measured characteristics, we also observed differences in subjective measures of quality of life that are sensitive to physical activity [34]. As a patient-reported outcome, quality of life provides a subjective measure of well-being [56] that is not only useful as an endpoint for medical decision making, but can also predict long-term survival in women [75, 76]. Quality of life is negatively impacted by chronic disease and obesity [77, 78], and physical activity can improve quality of life in both healthy women and those with chronic disease [79–81]. Differences in both the SF-36 physical function subscale and the Vitality Plus Scale support the validity of the single question. Specifically, the difference in physical function scores we observed between *not active* and *very active* women is equivalent to the 20-point difference previously observed in adults who did and did not meet the recommendation for at least 150 min/week of moderate physical activity [82]. Furthermore, the predicted increase in physical function score of 6 points with a one-level increase in habitual physical activity exceeds the mean difference in physical function scores of 2.74 points calculated to be due to long-term exercise interventions in women [81]. Hence, we believe the single question discriminates clinically meaningful differences in quality of life related to habitual physical activity levels.

Although not as widely used as the SF-36, the Vitality Plus Scale was initially validated against the SF-36 and found to have significant associations with the physical function subscale and, in particular, participation in vigorous and moderate activities [57]. Vitality Plus Scale scores also have a positive relationship with walking speed [57], which has been confirmed to agree with accelerometry measured gait speed in women [83]. No minimal clinically important difference has been established for the Vitality Plus Scale, but the predicted increase of 3.2 points for a one-level increase in habitual physical activity approximates the general recommendation for calculating minimally important differences in quality of life based on 0.5 SD in scores [84]. In our sample, the standard deviation for Vitality Plus Scores was 6.9, half of which would be 3.45 points. If applied to our sample as the minimally important difference, the difference in scores between *not active* and all other habitual activity levels exceeds that standard and reflects good ability of the single question to discriminate meaningful differences in activity-related quality of life between different levels of habitual physical activity.

### Limitations and strengths

There are clear limitations to our study as well as strengths. Primarily, objective measurement for determination of criterion validity is needed. Criterion validity provides the strongest evidence that a tool measures what it is intended to measure. Although we used multiple outcome variables that have previously been validated with accelerometry, direct validation of the single question with accelerometry as the criterion measure is needed and should be the focus of future research. Larger samples are also needed in addition to more diversity including both gender and race. However, not only did our sample exceed the calculated minimum size needed for the study, but the majority of correlations we observed for convergent validity were significant at the *p* <0.001 level and the majority of differences between habitual physical activity levels we observed for known-groups validity were significant at the *p* <0.001 level. Hence, we believe our sample of 120 women was adequate for validation. Furthermore, it included a remarkably wide range of ages that demonstrated no effect of age on the responses, which enhances the utility of the single question. Nevertheless, to our knowledge, the single question has been validated only in women, which greatly limits its generalizability. Although evidence is limited, the accuracy of self-reported activity can be influenced by gender, with women being less accurate than men [85]. Also, the women in the current and previous [49] validation studies were predominantly White, which further limits generalizability, especially as non-White adults and those from lower socioeconomic communities are less likely to meet physical activity guidelines compared to White adults [86, 87]. This underscores the need for establishing criterion validity as discussed above.

We recognize that although the correlations we observed between habitual physical activity levels and objective and subjective outcome variables were statistically significant, they were modest and somewhat weaker than the correlations previously reported for the same variables [49]. This may be due to differences in study designs. The previously reported study used a sample of older women that were restricted in age to ≥ 60 years and who had a notably greater average age of 73.7 years. Although the average age of our sample was 60.2 years, the actual range in age was 25–89 years. This may in some way have influenced the relationships we observed. Furthermore, the current study used the IPAQ-SF to calculate MET-min/week, while the previous study used a simple self-report of number of hours spent in light, moderate, and vigorous activities in an average week. This difference may also have influenced differences in the strength of the correlations. Nevertheless, we believe our use of linear regression to demonstrate predicted changes in outcome variables with increased levels of self-reported habitual physical activity offers additional support for the strength of the correlations as well as valuable perspective on use of the single question. Despite what we believe are strengths of our study, we recognize that the lack of pre-specified hypotheses is a weakness in our design.

Another limitation includes lack of prior qualitative validation of the question wording. Use of “lifestyle” rather than “physical activity” may influence interpretation of the question and what it is asking. However, we believe that use of “lifestyle” is an advantage of this question due to respondents’ difficulty categorizing and quantifying “activity” specifically in relation to intensity, frequency, and duration [43, 44]. Anecdotally, during data collection, we have observed women to struggle for prolonged periods of time when completing questionnaires such as the International Physical Activity Questionnaire (IPAQ) that asks them to not only categorize activity by intensity, but also quantify both frequency and duration. Since the clinical importance of physical activity is related to its outcomes rather than the activity per se and the single question has been found to be significantly related to those outcomes, we believe that use of the single question is appropriate. Nevertheless, qualitative research regarding the interpretation of the single question by respondents has not been done to our knowledge. A cognitive debriefing study could provide valuable insight regarding its perceived meaning and is recommended.

A final limitation is the potential influence of bias related to self-report. Observational research that collects data through self-report includes the risk of social desirability and recall bias [88]. Due to the widespread recognition of the benefits of physical activity, respondents may interpret being physically active as socially desirable, leading them to overreport their activity levels. However, evidence regarding the effect of social desirability bias on self-reported physical activity is specifically related to the need to quantify frequency (number of days and/or number of hours) [89, 90], which the single question does not ask respondents to do. Recall bias may also affect research findings through errors in recalling the specific type or quantity of activity. Here again, we believe that one of the strengths of the single question is that it uses categorical descriptors instead of asking respondents to quantify their activity into a specific number of days and/or hours. Nevertheless, we recognize that the risk of bias cannot be overlooked. The recommended strategy for overcoming bias in self-report is external validation [88]. We cannot overemphasize the need for future studies to establish criterion validity with accelerometry.

## Conclusion

A single question with categorical descriptors provides a clinically meaningful assessment of habitual physical activity in women across a wide age range. The four response options (*not active*,* somewhat active*,* active*,* very active*) accurately discriminate between different physical activity levels. Although further research is evidently needed, we believe our preliminary results provide support for use of the single question in clinical settings where brief, accurate assessment is needed.

## Data Availability

The dataset used and/or analyzed during the current study is available from the corresponding author on reasonable request.

## Abbreviations

BMI: Body mass index
IPAQ: International Physical Activity Questionnaire
IPAQ-SF: International Physical Activity Questionnaire – Short Form
MET: Metabolic equivalent
SF-36: Short Form-36
